# Automated Lesion and Feature Extraction Pipeline for Brain MRIs with Interpretability

**DOI:** 10.1007/s12021-024-09708-z

**Published:** 2025-01-09

**Authors:** Reza Eghbali, Pierre Nedelec, David Weiss, Radhika Bhalerao, Long Xie, Jeffrey D. Rudie, Chunlei Liu, Leo P. Sugrue, Andreas M. Rauschecker

**Affiliations:** 1 Department of Radiology and Biomedical Imaging, University of California, San Francisco, San Francisco, CA USA; 2 Department of Electrical Engineering and Computer Sciences, University of California, Berkeley, Berkeley, CA USA; 3 Berkeley Institute for Data Science, University of California, Berkeley, Berkeley, CA USA; 4 Department of Radiology, University of California, San Diego, San Diego, CA USA; 5 Department of Biomedical Engineering, Georgia Institute of Technology, Atlanta, GA USA; 6 Siemens Healthineers, Erlangen, Germany; 7 Helen Wills Neuroscience Institute, University of California, Berkeley, CA USA

**Keywords:** Neuroradiology, MRI pipeline, Radiomics

## Abstract

This paper introduces the Automated Lesion and Feature Extraction (ALFE) pipeline, an open-source, Python-based pipeline that consumes MR images of the brain and produces anatomical segmentations, lesion segmentations, and human-interpretable imaging features describing the lesions in the brain. ALFE pipeline is modeled after the neuroradiology workflow and generates features that can be used by physicians for quantitative analysis of clinical brain MRIs and for machine learning applications. The pipeline uses a decoupled design which allows the user to customize the image processing, image registrations, and AI segmentation tools without the need to change the business logic of the pipeline. In this manuscript, we give an overview of ALFE, present the main aspects of ALFE pipeline design philosophy, and present case studies.

**Supplementary Information:**

The online version contains supplementary material available at 10.1007/s12021-024-09708-z.

## Introduction

Magnetic resonance imaging (MRI) is widely used for clinical diagnosis and monitoring of brain conditions such as brain tumors, infections, inflammatory or demyelinating processes, vascular conditions, and degenerative diseases. Medical images such as MRIs constitute a vast repository of valuable data in the healthcare system, yet these data are not used to their full potential in improving healthcare delivery or further understanding of disease, generally only being archived for future use for the same patient’s care. In short, despite the emergence of many data science techniques that can utilize imaging data for novel insights, the vast majority of medical images are seen as pictures, not as data (Gillies et al., 2016). The reasons for this situation are numerous, including technical challenges for taking advantage of these data, such as their large dimensionality. Indeed, each brain MRI consists of multiple sequences (also called modalities or contrasts), each with approximately a million or more voxels. Classical dimensionality reduction techniques typically fail to capture clinically significant features. While modern learning algorithms may be amenable to performing tasks such as segmentation on these datasets, other tasks including diagnosis require some sort of dimensionality reduction techniques. Radiologists perform such dimensionality reduction on every MRI they read. They break the massive number of data points into discrete chunks by way of (1) identifying abnormalities, and then (2) describing those abnormalities along a set of pre-defined dimensions referred to as “imaging features”. These imaging features might include descriptors of lesion locations, signal properties, or volumetric and morphometric properties. Finally, these imaging features are used to inform an impression, or overall assessment.

**Fig. 1:**
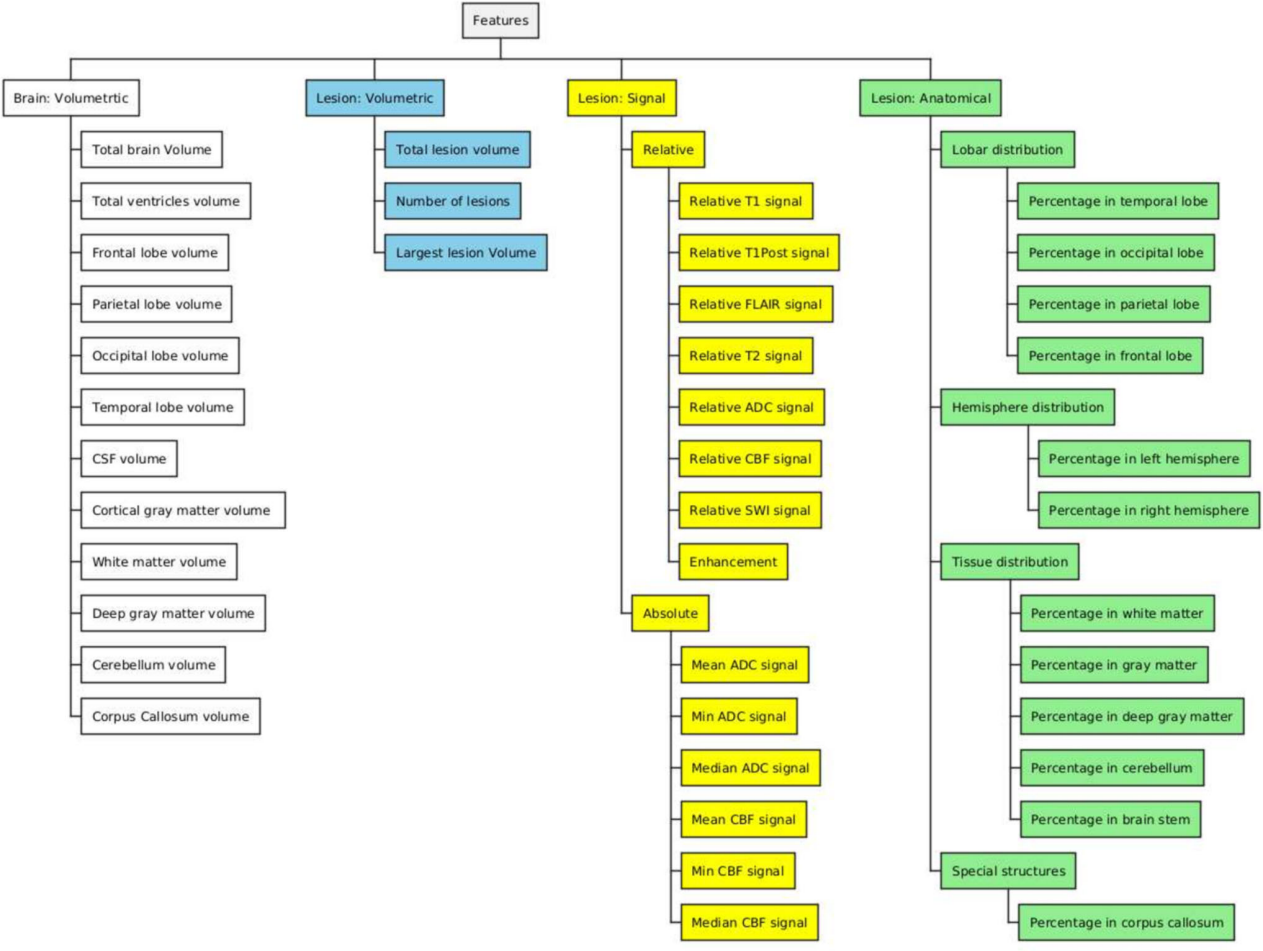
ALFE features. ALFE generates general volumetric features and features related to lesions that are found in the brain. The lesion features are subdivided into volumetric, signal, and anatomical features

**Fig. 2:**
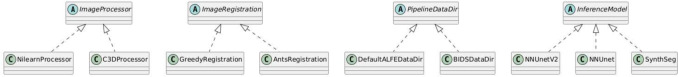
Class Diagram for ALFE

In the last decade, there has been an exponential increase in the use of radiomics and texture imaging features from Brain MRIs in training predictive diagnostic and prognostic models (Calabrese et al., 2022; Curtin et al., 2021; Destito et al., 2023; Fathi Kazerooni et al., 2022; Rauschecker et al., 2020; Rudie et al., 2020). Radiomics are usually extracted from tumor or lesion segmentations and capture geometric shape, size, pixel intensities, and inter-pixel relationships (Parekh & Jacobs, 2016). To extract such features, one needs to apply several processing and curation techniques to the images. These steps may include co-registration, resampling, segmentation, and feature extraction. In this work, we introduce an open-source, end-to-end automated lesion and feature extraction pipeline (ALFE), that performs preprocessing, registration, segmentation, and feature extraction tasks on MRI scans of adult brains independent of the underlying pathology. The extracted features include common radiomic features important for research, as well as more clinically-relevant features of interest to radiologists and clinicians. ALFE is a modular and extendable pipeline that generates human interpretable features, closely mimicking the features that expert radiologists use to guide them through diagnosis and progress assessment. In fact, the roots of this pipeline go back to the efforts started in Rauschecker et al. (2020); Rudie et al. (2020) on differential diagnosis.

**Fig. 3:**
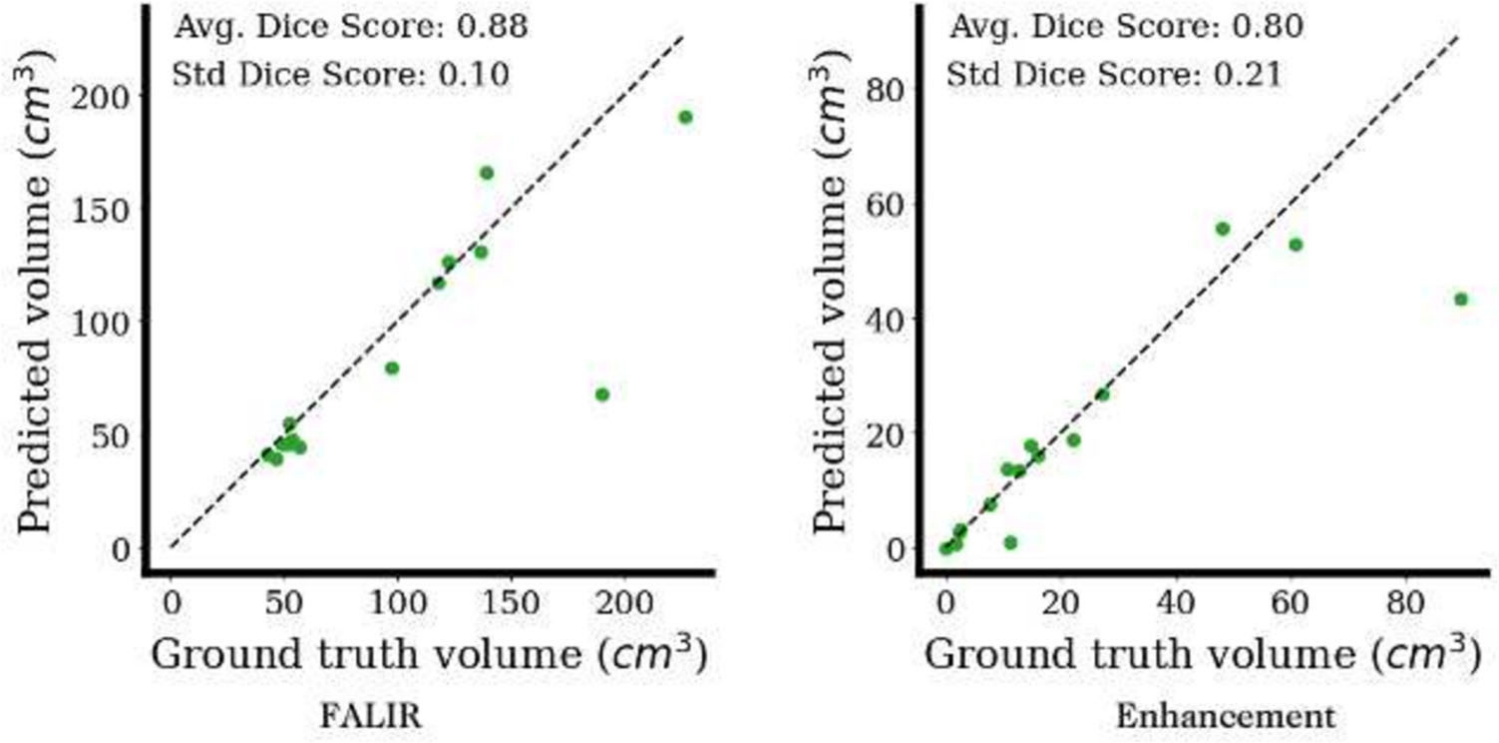
The test performance of ALFE’s default FLAIR and enhancement (T1Post) segmentation models. The test set includes manually segmented FLAIR and T1Post images for 5 high-grade glioma, 5 low-grade glioma and 5 primary CNS Lymphoma patients

**Fig. 4:**
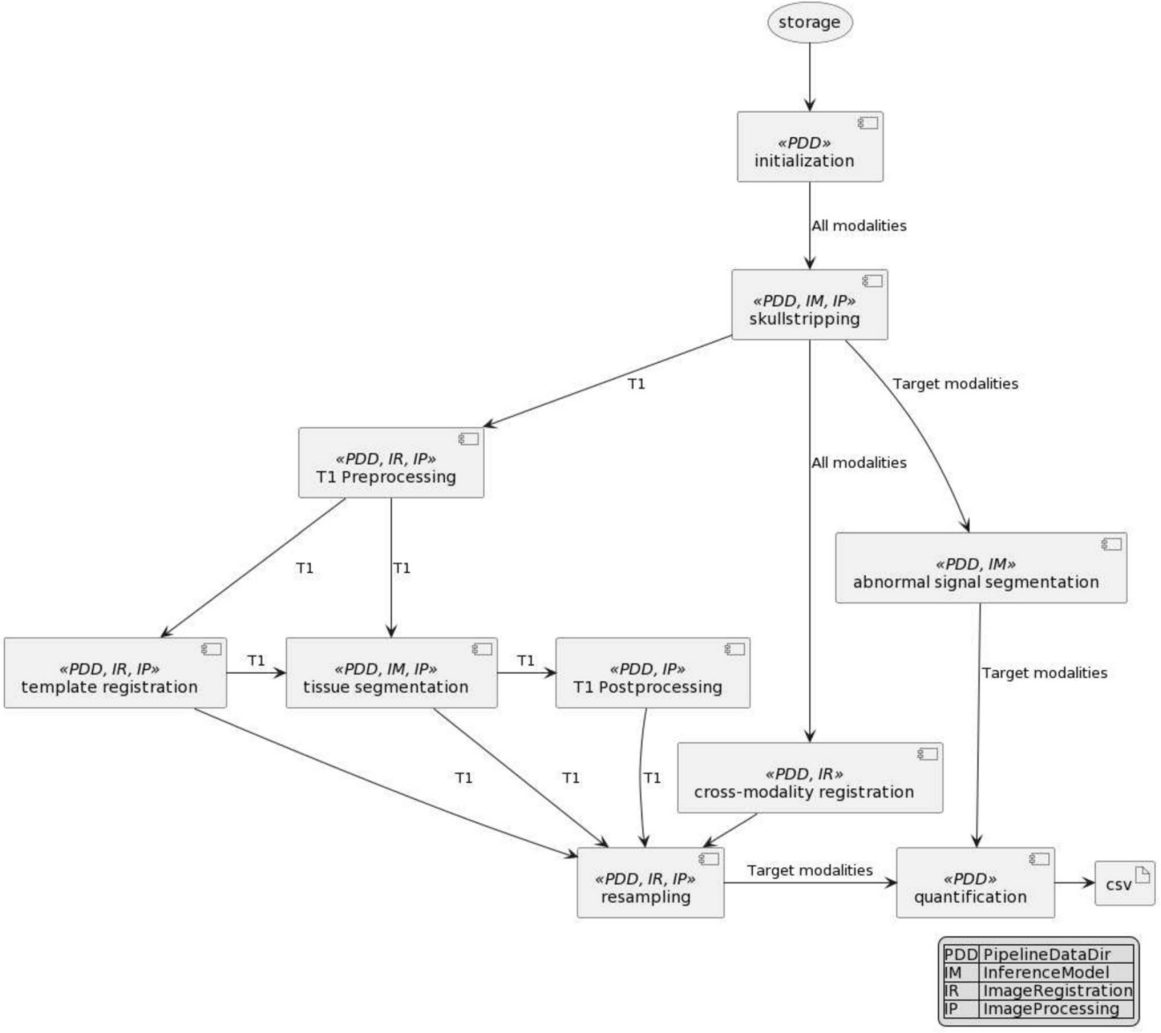
Pipeline block diagram. The pipeline consists of several tasks. Each task processes one or more sequences and depends on implementations of one or more classes discussed in Section “Decoupled Design”

The source code for the pipeline is available at https://github.com/reghbali/pyalfe released under BSD-3 license. The documentation is available at https://reghbali.github.io/pyalfe.

**Table 1:** Time, memory usage, and available modalities for each of the 6 cases

	Time (min.)	Memory (Gb)	T1	T1Post	FLAIR	T2	ADC	SWI	CBF
GBM	10	3.7	$*$	$*$	$*$	$*$			
PCNSL	36	10.4	$*$	$*$	$*$	$*$	$*$	$*$	$*$
METS	20	7.2	$*$	$*$	$*$	$*$	$*$		
TMS	14	4.5	$*$	$*$	$*$	$*$	$*$		
ALD	52	17.6	$*$	$*$	$*$	$*$	$*$	$*$	
BA	55	19.2	$*$	$*$	$*$	$*$	$*$	$*$	

Memory usage is the maximum resident set size reported by the GNU time command. The pipeline was run on a Linux machine with an Intel Xeon Gold 6234 CPU running at 3.30 GHz clock frequency and an Nvidia Titan RTX GPU

**Fig. 5:**
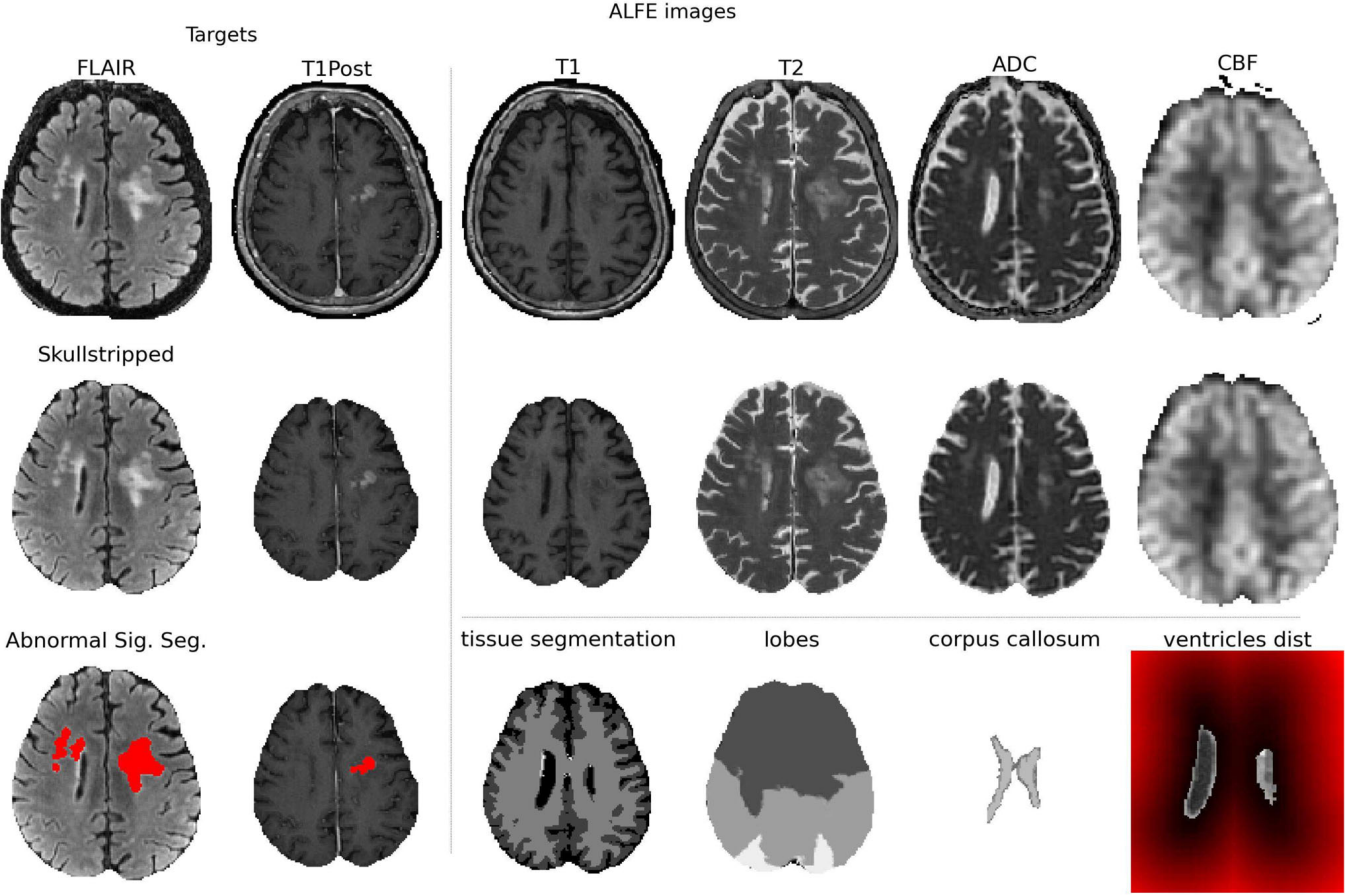
Input images (top row) and ALFE output images (middle and bottom rows) for a primary CNS lymphoma patient

### Related Work

In recent years multiple frameworks and projects have been developed to help researchers with various aspects of extracting radiomics. For instance, the pyradiomics project (Van Griethuysen et al., 2017) is, an open-source, Python-based tool for extraction of radiomics features from segmented tumors. Pyradiomics can be compared to the quantification stage which is the final stage of ALFE, with at least three notable differences: (1) ALFE features are generated from all the available MR sequences, while Pyradiomics only generates features from a single sequence that was used to define the lesion. (2) ALFE features include whole-brain descriptors not directly related to a lesion but nevertheless potentially important to clinicians in describing a brain MRI, such as ventricular volume. (3) ALFE and pyradiomic features of lesions differ, with pyradiomics extracting overall many more features. Indeed, pyradiomics produces a large set of geometric features which can complement ALFE features. For this reason, we have created the option for the user to install ALFE with pyradiomics support.

**Fig. 6:**
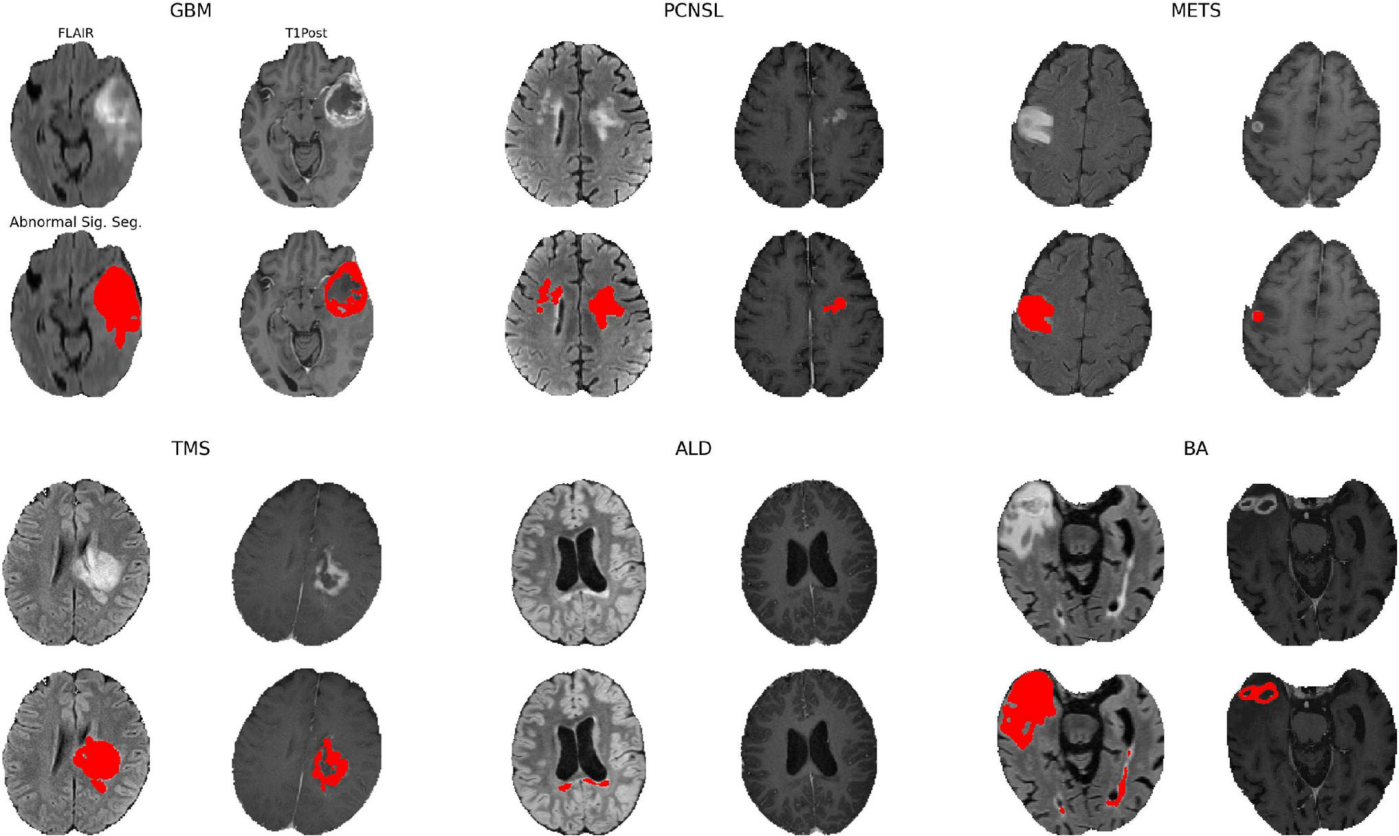
Target modalities and the segmentation of areas of abnormal signal produced by ALFE for all the 6 cases

Nipype is another project that has been developed to help researchers create neuroimaging software  (Gorgolewski et al., 2011). Nipype provides interfaces for many well-known neuroimaging tools. It also includes a framework for designing imaging processing workflows. We have taken inspiration from Nipype workflows and interfaces in the design of our pipeline. However, ALFE does not depend on Nipype because of the limitations Nipype would have imposed for our purposes and our desire to minimize the dependencies of our project.

Finally, the open source software suite FreeSurfer is widely used for skull-stripping, bias field correction, registration, and anatomical segmentation of Brain MRIs (Fischl, 2012). This software suite uses traditional image analysis algorithms, but recently, a robust contrast-agnostic deep learning method for tissue segmentation and cortex parcellation named SynthSeg has been added (Billot et al., 2023). ALFE supports SynthSeg for tissue segmentation. Thanks to the decoupled design of the pipeline, SynthSeg can easily replace the nnUnet (Isensee et al., 2021) trained for tissue segmentation, which is the default tissue segmentation method in ALFE.

## Pipeline Design

### Modeling the Neuroradiology Workflow

While interpreting MR images, a neuroradiologist must identify abnormalities usually relying on one or a few modalities of MRI. After identification of these abnormalities, various findings related to these abnormal regions are extracted such as the size of the abnormal regions, its anatomical location (e.g. the lobar location, white vs. gray matter lesion), and signal levels of other modalities of MRI over the abnormal region (e.g. ADC values over the enhancing lesion that can indicate restricted diffusion). These findings are used by neuroradiologists to produce an assessment of these abnormalities.

ALFE is designed to replicate this workflow. The user designates an MRI pulse sequence as the target modality. The target modalities can be Fluid Attenuated Inversion Recovery (FLAIR) and/or T1 post-contrast (T1Post). Segmentation of areas of abnormal signal is then performed on this imaging sequence. Afterwards, various features are extracted. Examples of features include size of the abnormal regions (e.g. total enhancing lesion volume), anatomical location (e.g. percentage of abnormality in frontal lobes), and signal levels of other modalities of MRI over the abnormal regions (e.g. Minimum ADC value over the enhancing lesions). These features can then be a helpful adjunct to clinical assessment providing quantitative values in an automated workflow, or they can be used in an ML model trained on diagnostic or prognostic targets.

### Human Interpretable Features

ALFE generates human interpretable brain volumetric and lesion features. The lesion features can be categorized into three groups: signal, anatomical, and volumetric (see Fig. 1):

#### Brain Volumetric Features

These set of features include lobe volumes, ventricular volumes, tissue volume, and total brain volume.

#### Lesion Volumetric Features

These set of features include the total lesion volume, the number of detected lesions, and a vector of individual lesion volumes.

#### Lesion Signal Features

These features measure the average signal for each pulse sequence on the lesion relative to the signal averaged over the healthy tissue of the same type (white matter, gray matter, deep gray matter, or cerebellum). The tissue type is either provided by the user or set to “auto” in which case the tissue type that contains the majority of the volume of the lesion is automatically selected. For instance relative T1 signal on the FLAIR lesion is defined as:

$$\begin{aligned} \text {relative T1 signal} = \frac{\text {avg. T1 signal over lesion}}{\text {avg. T1 signal over healthy tissue}}. \end{aligned}$$

We also calculate the amount of enhancement over the lesion as:

$$\begin{aligned} \text {enhancement} = \frac{\text {avg. T1Post signal over lesion}}{\text {avg. T1 signal over lesion}}. \end{aligned}$$

Here, T1Post refers to post-contrast enhanced T1-weighted image. Another set of signal features measure the absolute signal statistics such as mean, min, and median for sequences where signal has a unit such as Apparent Diffusion Coefficient (ADC) and Cerebral Blood Flow (CBF).

**Table 2:** Brain volumetric features for the PCNSL case

Feature	value
total brain volume $(\textrm{mm}^3)$	1248096
total ventricles volume $(\textrm{mm}^3)$	36630
volume of background $(\textrm{mm}^3)$	3342244
volume of csf $(\textrm{mm}^3)$	312965
volume of cortical gray matter $(\textrm{mm}^3)$	421988
volume of white matter $(\textrm{mm}^3)$	422067
volume of deep gray matter $(\textrm{mm}^3)$	36505
volume of brain stem $(\textrm{mm}^3)$	22320
volume of cerebellum $(\textrm{mm}^3)$	147941
volume of Frontal $(\textrm{mm}^3)$	498876
volume of Parietal $(\textrm{mm}^3)$	247690
volume of Occipital $(\textrm{mm}^3)$	148004
volume of Temporal $(\textrm{mm}^3)$	219727
volume of AnteriorTemporal $(\textrm{mm}^3)$	69042
volume of MiddleTemporal $(\textrm{mm}^3)$	110651
volume of PosteriorTemporal $(\textrm{mm}^3)$	40034
volume of Parietal Occipital $(\textrm{mm}^3)$	395694
volume of CorpusCallosum $(\textrm{mm}^3)$	24075
volume of CorpusCallosum Rostrum $(\textrm{mm}^3)$	4485
volume of CorpusCallosum Genu $(\textrm{mm}^3)$	5544
volume of CorpusCallosum Body $(\textrm{mm}^3)$	8100
volume of CorpusCallosum Isthmus $(\textrm{mm}^3)$	2429
volume of CorpusCallosum Splenium $(\textrm{mm}^3)$	3517
volume of CSF $(\textrm{mm}^3)$	286888
volume of Cortical Gray Matter $(\textrm{mm}^3)$	427156
volume of White Matter $(\textrm{mm}^3)$	423359
volume of Deep Gray Matter $(\textrm{mm}^3)$	40768
volume of Brain Stem $(\textrm{mm}^3)$	21866
volume of Cerebellum $(\textrm{mm}^3)$	131125

#### Lesion Anatomical Features

These features measure the overlap of the lesions with brain hemispheres, lobes, different tissue types, and various structures such as cerebellum, brain stem, or corpus callosum.

**Table 3:** Summary lesion features for the PCNSL case

Feature	T1Post Lesion	FLAIR Lesion
total lesion volume $(\textrm{mm}^3)$	1619.0	16857.3
lesion volume in csf $(\textrm{mm}^3)$	0	253.5
lesion volume in cortical gray matter $(\textrm{mm}^3)$	4.0	732.0
lesion volume in white matter $(\textrm{mm}^3)$	1615.0	15678.3
lesion volume in deep gray matter $(\textrm{mm}^3)$	0	146.8
lesion volume in brain stem $(\textrm{mm}^3)$	0	0
lesion volume in cerebellum $(\textrm{mm}^3)$	0	0
relative T1 signal	0.9	0.9
relative T1Post signal	1.5	1.0
relative FLAIR signal	1.3	1.5
relative T2 signal	1.0	1.2
relative ADC signal	0.9	1.0
mean ADC signal $(10^{-6} \textrm{mm}^2/\textrm{s})$	845.8	936.5
median ADC signal $(10^{-6} \textrm{mm}^2/\textrm{s})$	800.4	870.7
five percentile ADC signal $(10^{-6} \textrm{mm}^2/\textrm{s})$	638.4	674.4
ninety five percentile ADC signal $(10^{-6} \textrm{mm}^2/\textrm{s})$	1175.3	1391.4
relative SWI signal	1.0	1.1
relative CBF signal	1.1	0.8
mean CBF signal $(\textrm{mL}/100 \textrm{g}/\textrm{min})$	64.7	48.9
median CBF signal $(\textrm{mL}/100 \textrm{g}/\textrm{min})$	66.6	47.8
five percentile CBF signal $(\textrm{mL}/100 \textrm{g}/\textrm{min})$	44.1	29.4
ninety five percentile CBF signal $(\textrm{mL}/100 \textrm{g}/\textrm{min})$	79.5	72.9
enhancement	1.9	1.3
average dist to ventricles (voxels)	9.1	9.7
minimum dist to Ventricles (voxels)	0.9	0
lesion volume in Frontal $(\textrm{mm}^3)$	1619.0	14613.0
percentage volume in Frontal	100.0	86.7
lesion volume in Parietal $(\textrm{mm}^3)$	0	1543.8
percentage volume in Parietal	0	9.2
lesion volume in Occipital $(\textrm{mm}^3)$	0	480.8
percentage volume in Occipital	0	2.9
lesion volume in Temporal $(\textrm{mm}^3)$	0	219.8
percentage volume in Temporal	0	1.3
lesion volume in CorpusCallosum $(\textrm{mm}^3)$	196.0	1187.0
percentage volume in CorpusCallosum	12.1	7.0
number of lesions	1	30
largest lesion volume $(\textrm{mm}^3)$	1619.0	10418.8

**Table 4:** Individual lesion features for the four largest FLAIR lesions in the PCNSL case

Feature	Lesion 0	Lesion 1	Lesion 2	Lesion 3
total lesion volume $(\textrm{mm}^3)$	10418.8	3280.0	955.0	493.5
lesion volume in csf $(\textrm{mm}^3)$	101.5	86.3	40.0	0
lesion volume in cortical gray matter $(\textrm{mm}^3)$	201.5	392.8	54.0	38.0
lesion volume in white matter $(\textrm{mm}^3)$	9998.3	2758.0	832.0	455.5
lesion volume in deep gray matter $(\textrm{mm}^3)$	117.5	27.3	0	0
lesion volume in brain stem $(\textrm{mm}^3)$	0	0	0	0
lesion volume in cerebellum $(\textrm{mm}^3)$	0	0	0	0
relative T1 signal	0.9	0.9	0.9	0.9
relative T1Post signal	1.0	0.9	0.9	0.9
relative FLAIR signal	1.5	1.5	1.8	1.8
relative T2 signal	1.2	1.2	1.4	1.4
relative ADC signal	1.0	1.0	1.1	1.1
mean ADC signal $(10^{-6} \textrm{mm}^2/\textrm{s})$	921.7	930.8	1064.5	1062.2
median ADC signal $(10^{-6} \textrm{mm}^2/\textrm{s})$	863.6	882.9	963.2	1072.0
five percentile ADC signal $(10^{-6} \textrm{mm}^2/\textrm{s})$	662.7	711.9	772.0	718.1
ninety five percentile ADC signal $(10^{-6} \textrm{mm}^2/\textrm{s})$	1409.5	1261.1	1719.5	1354.2
relative SWI signal	1.1	1.1	1.1	1.1
relative CBF signal	0.9	0.7	0.5	0.6
mean CBF signal $(\textrm{mL}/100 \textrm{g}/\textrm{min})$	53.0	40.5	32.0	36.7
median CBF signal $(\textrm{mL}/100 \textrm{g}/\textrm{min})$	51.5	38.5	31.2	36.3
five percentile CBF signal $(\textrm{mL}/100 \textrm{g}/\textrm{min})$	35.6	26.5	26.4	30.8
ninety five percentile CBF signal $(\textrm{mL}/100 \textrm{g}/\textrm{min})$	74.4	62.2	40.8	44.0
enhancement	1.3	1.2	1.2	1.1
average dist to ventricles (voxels)	10.2	8.3	4.2	3.1
minimum dist to Ventricles (voxels)	0	0	0	0.5
lesion volume in Frontal $(\textrm{mm}^3)$	10309.8	3280.0	0	0
percentage volume in Frontal	99.0	100.0	0	0
lesion volume in Parietal $(\textrm{mm}^3)$	109.0	0	709.5	146.0
percentage volume in Parietal	1.0	0	74.3	29.6
lesion volume in Occipital $(\textrm{mm}^3)$	0	0	197.5	205.5
percentage volume in Occipital	0	0	20.7	41.6
lesion volume in Temporal $(\textrm{mm}^3)$	0	0	48.0	142.0
percentage volume in Temporal	0	0	5.0	28.8
lesion volume in CorpusCallosum $(\textrm{mm}^3)$	930.5	158.5	74.3	21.8
percentage volume in CorpusCallosum	8.9	4.8	7.8	4.4

**Table 5:** Summary lesion features for the GBM case

Feature	T1Post Lesion	FLAIR Lesion
total lesion volume $(\textrm{mm}^3)$	29146	112314
lesion volume in csf $(\textrm{mm}^3)$	2179	4783
lesion volume in cortical gray matter $(\textrm{mm}^3)$	17740	56389
lesion volume in white matter $(\textrm{mm}^3)$	9227	49929
lesion volume in deep gray matter $(\textrm{mm}^3)$	0	1173
lesion volume in brain stem $(\textrm{mm}^3)$	0	0
lesion volume in cerebellum $(\textrm{mm}^3)$	0	0
relative T1 signal	1.0	1.0
relative T1Post signal	1.5	1.0
relative FLAIR signal	1.7	1.8
relative T2 signal	1.8	2.4
enhancement	1.9	1.3
average dist to ventricles (voxels)	32.7	27.0
minimum dist to Ventricles (voxels)	8.1	0
lesion volume in Frontal $(\textrm{mm}^3)$	5603	24311
percentage volume in Frontal	19.2	21.6
lesion volume in Parietal $(\textrm{mm}^3)$	1909	10414
percentage volume in Parietal	6.5	9.3
lesion volume in Occipital $(\textrm{mm}^3)$	886	2588
percentage volume in Occipital	3.0	2.3
lesion volume in Temporal $(\textrm{mm}^3)$	20631	74714
percentage volume in Temporal	70.8	66.5
lesion volume in CorpusCallosum $(\textrm{mm}^3)$	0	8
percentage volume in CorpusCallosum	0	0.0
number of lesions	1	2
largest lesion volume $(\textrm{mm}^3)$	29146	112229

**Table 6:** Summary lesion features for the METS case

Feature	T1Post Lesion	FLAIR Lesion
total lesion volume $(\textrm{mm}^3)$	797.8	17631.4
lesion volume in csf $(\textrm{mm}^3)$	130.8	2370.9
lesion volume in cortical gray matter $(\textrm{mm}^3)$	571.4	10392.6
lesion volume in white matter $(\textrm{mm}^3)$	95.6	4867.9
lesion volume in deep gray matter $(\textrm{mm}^3)$	0	0
lesion volume in brain stem $(\textrm{mm}^3)$	0	0
lesion volume in cerebellum $(\textrm{mm}^3)$	0	0
relative T1 signal	0.7	0.8
relative T1Post signal	1.3	0.8
relative FLAIR signal	1.7	1.9
relative T2 signal	0.9	1.6
relative ADC signal	1.4	1.5
mean ADC signal $(10^{-6} \textrm{mm}^2/\textrm{s})$	1278.9	1328.8
median ADC signal $(10^{-6} \textrm{mm}^2/\textrm{s})$	1545.4	1390.6
five percentile ADC signal $(10^{-6} \textrm{mm}^2/\textrm{s})$	22.9	119.3
ninety five percentile ADC signal $(10^{-6} \textrm{mm}^2/\textrm{s})$	2568.3	2000.9
enhancement	1.9	1.0
average dist to ventricles (voxels)	64.5	50.7
minimum dist to Ventricles (voxels)	49.5	0
lesion volume in Frontal $(\textrm{mm}^3)$	479.4	12222.7
percentage volume in Frontal	60.1	69.3
lesion volume in Parietal $(\textrm{mm}^3)$	286.9	4849.7
percentage volume in Parietal	36.0	27.5
lesion volume in Occipital $(\textrm{mm}^3)$	31.5	557.9
percentage volume in Occipital	3.9	3.2
lesion volume in Temporal $(\textrm{mm}^3)$	0	0
percentage volume in Temporal	0	0
lesion volume in CorpusCallosum $(\textrm{mm}^3)$	0	0
percentage volume in CorpusCallosum	0	0
number of lesions	2	4
largest lesion volume $(\textrm{mm}^3)$	766.4	16549.9

**Table 7:** Summary lesion features for the Brain Abscess case

Feature	T1Post Lesion	FLAIR Lesion
total lesion volume $(\textrm{mm}^3)$	3089.2	57528.4
lesion volume in csf $(\textrm{mm}^3)$	0	99.1
lesion volume in cortical gray matter $(\textrm{mm}^3)$	1298.2	27092.0
lesion volume in white matter $(\textrm{mm}^3)$	1791.0	29537.4
lesion volume in deep gray matter $(\textrm{mm}^3)$	0	783.6
lesion volume in brain stem $(\textrm{mm}^3)$	0	0
lesion volume in cerebellum $(\textrm{mm}^3)$	0	0
relative T1 signal	0.7	0.7
relative T1Post signal	1.9	0.8
relative FLAIR signal	1.8	1.8
relative T2 signal	1.1	1.4
relative ADC signal	0.8	1.1
mean ADC signal $(10^{-6} \textrm{mm}^2/\textrm{s})$	1200.2	1508.3
median ADC signal $(10^{-6} \textrm{mm}^2/\textrm{s})$	1218.3	1407.5
five percentile ADC signal $(10^{-6} \textrm{mm}^2/\textrm{s})$	585.1	533.8
ninety five percentile ADC signal $(10^{-6} \textrm{mm}^2/\textrm{s})$	1792.6	3363.7
relative SWI signal	1.0	1.1
enhancement	3.3	1.3
average dist to ventricles (voxels)	38.4	38.5
minimum dist to Ventricles (voxels)	14.8	0.0
lesion volume in Frontal $(\textrm{mm}^3)$	0	8506.7
percentage volume in Frontal	0	14.8
lesion volume in Parietal $(\textrm{mm}^3)$	0	1127.9
percentage volume in Parietal	0	2.0
lesion volume in Occipital $(\textrm{mm}^3)$	0	2850.5
percentage volume in Occipital	0	5.0
lesion volume in Temporal $(\textrm{mm}^3)$	3089.2	45043.2
percentage volume in Temporal	100.0	78.3
lesion volume in CorpusCallosum $(\textrm{mm}^3)$	0	252.9
percentage volume in CorpusCallosum	0	0.4
number of lesions	1	25
largest lesion volume $(\textrm{mm}^3)$	3089.2	50675.1

**Table 8:** Summary lesion features for the ALD case

Feature	T1Post Lesion	FLAIR Lesion
total lesion volume $(\textrm{mm}^3)$	0.000000	1191.9
lesion volume in csf $(\textrm{mm}^3)$	–	13.3
lesion volume in cortical gray matter $(\textrm{mm}^3)$	–	74.5
lesion volume in white matter $(\textrm{mm}^3)$	–	1104.1
lesion volume in deep gray matter $(\textrm{mm}^3)$	–	0
lesion volume in brain stem $(\textrm{mm}^3)$	–	0
lesion volume in cerebellum $(\textrm{mm}^3)$	–	0
relative T1 signal	–	1.0
relative T1Post signal	–	1.0
relative FLAIR signal	–	1.6
relative T2 signal	–	0.8
relative ADC signal	–	1.2
mean ADC signal $(10^{-6} \textrm{mm}^2/\textrm{s})$	–	759.4
median ADC signal $(10^{-6} \textrm{mm}^2/\textrm{s})$	–	738.8
five percentile ADC signal $(10^{-6} \textrm{mm}^2/\textrm{s})$	–	571.8
ninety five percentile ADC signal $(10^{-6} \textrm{mm}^2/\textrm{s})$	–	1050.1
relative SWI signal	–	1.0
enhancement	–	1.0
average dist to ventricles (voxels)	–	7.6
minimum dist to Ventricles (voxels)	–	0
lesion volume in Frontal $(\textrm{mm}^3)$	–	0
percentage volume in Frontal	–	0
lesion volume in Parietal $(\textrm{mm}^3)$	–	969.5
percentage volume in Parietal	–	81.3
lesion volume in Occipital $(\textrm{mm}^3)$	–	222.3
percentage volume in Occipital	–	18.7
lesion volume in Temporal $(\textrm{mm}^3)$	–	0
percentage volume in Temporal	–	0
lesion volume in CorpusCallosum $(\textrm{mm}^3)$	–	775.9
percentage volume in CorpusCallosum	–	65.1
number of lesions	0.000000	3
largest lesion volume $(\textrm{mm}^3)$	0.000000	690.3

**Table 9:** Summary lesion features for the TMS case

Feature	T1Post Lesion	FLAIR Lesion
total lesion volume $(\textrm{mm}^3)$	24421.1	38096.2
lesion volume in csf $(\textrm{mm}^3)$	2360.7	2818.7
lesion volume in cortical gray matter $(\textrm{mm}^3)$	3734.0	5799.9
lesion volume in white matter $(\textrm{mm}^3)$	17157.1	28470.5
lesion volume in deep gray matter $(\textrm{mm}^3)$	731.6	575.7
lesion volume in brain stem $(\textrm{mm}^3)$	0	0
lesion volume in cerebellum $(\textrm{mm}^3)$	0	0
relative T1 signal	0.9	0.9
relative T1Post signal	1.6	1.3
relative FLAIR signal	1.6	1.8
relative T2 signal	1.9	2.0
relative ADC signal	1.4	1.4
mean ADC signal $(10^{-6} \textrm{mm}^2/\textrm{s})$	1254.6	1211.6
median ADC signal $(10^{-6} \textrm{mm}^2/\textrm{s})$	1227.2	1184.0
five percentile ADC signal $(10^{-6} \textrm{mm}^2/\textrm{s})$	875.0	844.1
ninety five percentile ADC signal $(10^{-6} \textrm{mm}^2/\textrm{s})$	1729.4	1686.2
enhancement	1.3	1.1
average dist to ventricles (voxels)	11.6	15.0
minimum dist to Ventricles (voxels)	0	0
lesion volume in Frontal $(\textrm{mm}^3)$	10777.9	16757.8
percentage volume in Frontal	44.1	44.0
lesion volume in Parietal $(\textrm{mm}^3)$	10481.8	18954.3
percentage volume in Parietal	42.9	49.8
lesion volume in Occipital $(\textrm{mm}^3)$	396.3	378.3
percentage volume in Occipital	1.6	1.0
lesion volume in Temporal $(\textrm{mm}^3)$	2765.0	2005.8
percentage volume in Temporal	11.3	5.3
lesion volume in CorpusCallosum $(\textrm{mm}^3)$	2929.8	4724.9
percentage volume in CorpusCallosum	12.0	12.4
number of lesions	1	2
largest lesion volume $(\textrm{mm}^3)$	24421.1	38077.9

### Decoupled Design

A modern MRI pipeline needs to interact with a directory structure in the file system, perform image registration, machine learning inference, and various image pre- and post-processing tasks. A main design consideration for ALFE is that the business logic of the pipeline should be agnostic to the particular implementation of registration tool, image processing tool, machine learning inference models, and the directory structure used as long as they provide the required functionality for the pipeline. In object-oriented programming terminology, this is known as interface inheritance, which can be emulated in Python using abstract base classes (abc).

Figure 2 shows the main abstract classes that are used by the pipeline. ALFE provides a few implementations for each one of these abstract classes and has default implementations that allow the user to run the pipeline without the need to make any decision as to what implementations to use. The default choices listed above can be changed by the user to another implementation provided by ALFE or by the user. The four main abstract classes used in the design of the pipeline are ImageProcessing, ImageRegistration, PipelineDataDir, and InferenceModel.

#### ImageRegistration

includes affine and deformable registration methods. The default implementation in ALFE uses *Greedy* (Yushkevich et al., 2016). ALFE also provides a second implementation based on *ANTsPy*, a Python implementation of ANTs (Avants et al., 2011).

#### ImageProcessing

includes most of the common MRI processing methods such as masking, resampling, binary operation between two images, and finding largest connected component. The default implementation used by ALFE is the Python native library *Nilearn* (Abraham et al., 2014), while an alternative implementation is provided based on *Convert3D (C3D)* (Yushkevich et al., 2006).

#### PipelineDataDir

includes methods for reading and writing the input, output, intermediate images, and quantification files. The default implementation uses a basic directory hierarchy organized by study and sequences. ALFE also provides an implementation for working with the brain imaging data structure (BIDS) (Gorgolewski et al., 2016).

#### InferenceModel

has a method for prediction that is used to generate various segmentation maps. ALFE comes with a default implementation that wraps around nnUNet (Isensee et al., 2021) and uses models trained for skullstripping, tissue segmentation, FLAIR segmentation, and abnormal T1Post enhancing signal segmentation. We also provide an implementation using SynthSeg, available through FreeSurfer, which can be used as an alternative to the default model for tissue segmentation. Figure 3 shows the test performance of the default models for T1Post and FLAIR abnormal signal segmentation.

### Modular Design

The pipeline is organized into 8 tasks: initialization, skullstripping, T1 preprocessing, inter-modality registration, template registration, abnormal signal detection, tissue segmentation, resampling to target modality and quantification. Figure 4 shows the diagram of the pipeline. Each task may work on all or a subset of modalities and utilizes one or more of the components in Figure  2 discussed in section 2.3.

**Initialization** simply creates the corresponding modality directories in the process directory and copies the modality images from the input dir to their dedicated processed directory.

**Skullstripping** removes the skull by using a 3D UNet.

**Inter-modality registration** registers all modalities to target modalites via affine registration.

**T1 preprocessing** up-samples the image as needed and trims the neck.

**Template registration** registers various anatomical templates to the T1 image.

**Abnormal signal detection** detects the abnormal regions (tumors and lesions) in the target modality images.

**Tissue segmentation** segments white matter, gray matter, deep gray matter, cerebellum, CSF, and brainstem. The segmentation is performed by a 3D UNet that receives the preprocessed T1 image and a the template tissue segmentation transformed to patient’s T1 space as a prior. This approach was first proposed in Weiss et al. (2021), who observed that providing an atlas based prior improves the robustness of tissue segmentation.

**Resampling** transforms the output of template registration and tissue segmentation to the target space.

**Quantification** uses the generated lesion and tissue masks alongside registered images to produce a list of quantitative features, the final output of ALFE.

### User Interface

We designed the pipeline with ease of use in mind, particularly for clinical audiences. The pipeline can be installed using the Python package-management system, pip. After installation, the user has to run pyalfe download models to download the segmentation models and pyalfe config, which allows the user to configure the pipeline in a short interactive session. To run the pipeline, the user can execute pyalfe run ACCESSION, where ACCESSION is the name of the directory under which the imaging data for an MRI study is stored. Additionally, for users who prefer using Python scripts, the pipeline can be run by importing the pyalfe package in the Python script. All the configured options can be overwritten using the appropriate flags when running the pipeline. To get a list of options, the user can run pyalfe run –help. The user can also use the –no-overwrite flag to prevent the pipeline from overwriting output images that already exist. This option is useful if the pipeline was previously run but aborted and the user wants to skip the steps that were completed in the previous run. Another use case of this option is when the user has pre-computed certain pipeline outputs such as lesion segmentations and wishes to skip the corresponding tasks in the pipeline. This can be achieved by copying those pre-computed segmentation maps to the output directory and using the –no-overwrite flag.

## Case Studies

We applied ALFE to several diverse clinical MRI scans with different underlying diagnoses, including the following patients: a 62 year old man with primary central nervous system lymphoma (PCNSL), a 53 year old man with glioblastoma (GBM), a 57 year old woman with breast cancer metastatic to brain (METS), a 90 year old man with brain abscess (BA), a 27 year old woman with tumefactive multiple sclerosis (TMS), and a 10 year old boy with X-linked adrenoleukodystrophy (ALD). The MRI scan of a patient with glioblastoma (GBM) was obtained through the publicly available BraTS 2019 dataset (Menze et al., 2014; Bakas et al., 2017, 2018), and the MRI scan of the patient with metastatic cancer (METS) is publicly available through the UCSF-BMSR dataset (Rudie et al., 2023). The remainder of the cases were from our own institution’s imaging archives. To provide insight into the time and space complexity of the pipeline, we have recorded these details for all the cases in Table 1.

Figure 5 shows a subset of input images and output images generated by ALFE for the PCNSL patient and Fig. 6 shows the target modalities and the abnormal signal segmentation for all the patients.

ALFE generates a list of quantifiable features, which are shown as examples for the patient with PCNSL in Tables 2 and 3. These demonstrate the brain volumetric and summary lesion features, respectively. The features capturing the distance to the ventricles indicate the existence of periventricular lesions, which are common in PCNSL. The ADC signal features (e.g., mean ADC signal of T1Post Lesion of $799 \times 10^{-6} \textrm{mm}^2/\textrm{s}$) indicate restricted diffusion, which is an important feature for the differential diagnosis of PCNSL and also can serve as a prognostic indicator (Barajas et al., 2010).

To investigate the sensitivity of the generated features to the choice of image registration and processing tools, we also ran ALFE for the PCNSL case using non-default options: the image processing implementation based on C3D and the image registration implementation based on ANTsPy. The resulting features, provided in the Supplementary Materials, are very close to those presented here that were generated with the default options (Table 4).

Tables 5, 6, 7, 8, and 9 provide the summary lesion features generated by ALFE for the remaining cases. In the METS case (Table  6), there is a large overlap between the lesion and the gray matter for both FLAIR and T1Post lesions (78% of T1Post lesion and 63% of FLAIR lesion) consistent with propensity of METS for grey-white matter junction 1. Another notable feature is the FLAIR lesion enhancement which is 1.03 meaning that the average T1Post signal and average T1 signal over the FLAIR lesion are almost equal. In other words, much of the FLAIR lesion is nonenhancing, as it represents edema. In contrast, enhancement over the FLAIR lesion in the GBM case (Table 5) is 1.28, as much of the tumor demonstrates contrast enhancement, with only a small amount of true edema. In the ALD case, the features indicate one FLAIR lesion with more than 91% of its volume intersecting the white matter and around 66% its volume in the corpus callosum. The lesion only involves the parietal and occipital lobes out of the four lobes, which is consistent with the fact that in the majority of ALD patients, the lesion starts in the splenium of the corpus callosum and progresses to the adjacent parieto-occipital white matter (Van der Knaap & Valk, 2005).

## Discussion

The open-source end-to-end ALFE pipeline ingests and pre-processes clinical brain MRIs, identifies and segments areas of abnormality (lesions), and characterizes the brain and its lesions along a number of clinically useful feature dimensions. We posit that ALFE is a useful comprehensive tool for quantitative clinical brain MRI analysis.

This fully automated pipeline requires T1-weighted and FLAIR imaging sequences, although no specific acquisition parameters are required. ALFE is flexible with respect to additional imaging sequences, with the current version of ALFE taking into account T1 signal, T1 post-contrast enhancement, FLAIR, T2, ADC, SWI, and CBF. Since clinical MRI sequences are generally not quantitative, signal measurements are generally made with respect to non-lesional tissue. Where quantitative measurements are available (e.g. ADC), the pipeline reports these directly within a region of interest. Given the importance of anatomical location of lesions in the brain, a major focus of ALFE is to describe the presence of lesions with respect to anatomical regions, including lobes, hemispheres, deep gray matter, cerebellum, and brainstem. These anatomical features are in contrast to more common radiomic features extracted on the lesion itself irrespective of its anatomical location. Nevertheless, we acknowledge the importance of radiomic features, and therefore pyradiomics is incorporated into the output of ALFE. As a whole, ALFE offers an end-to-end automated pipeline for a comprehensive description of brain MRI clinical abnormalities.

### Potential Use Cases

We have demonstrated the utility of ALFE and its outputs with three examples of neoplastic clinical entities: GBM, PCNSL, and a metastatic tumor, and three examples of non-neoplastic processes: Brain Abscess, tumefactive multiple sclerosis, and ALD. These cases demonstrate that the pipeline is not specific to particular tumors or even to neoplastic processes. It is broadly applicable also to infectious, inflammatory, or other conditions causing brain lesions, including those that are very rare (e.g. ALD). Further, given the pipeline’s successful application to a case from outside our own institution, we believe that it will be relevant to data from other institutions. However, we note that it is beyond the scope of this manuscript to validate the pipeline for a large number of institutions and a nearly infinite number of possible image abnormalities. By making this tool and the associated models publicly available, we hope that other users will test the pipeline in the context of new use cases in new settings. Given the modular and customizable design philosophy, any components of the pipeline (e.g. the particular FLAIR segmentation model) can be adjusted. For example, a researcher could decide to optimize a segmentation model for a specific pathology (e.g. PCNSL) at their own institution, creating an single-institution-optimized PCNSL ALFE.

### Customizable Design Philosophy

One of the key strengths of the ALFE pipeline lies in its decoupled design philosophy. By decoupling image processing, image registrations, and AI segmentation tools from the core business logic of the pipeline, ALFE offers users a high degree of customization and flexibility. This design approach empowers researchers and clinicians to tailor the pipeline to their specific needs and preferences without sacrificing functionality or performance. Moreover, this flexibility ensures that we can adapt ALFE to evolving registration techniques, segmentation algorithms, and clinical requirements, making it a valuable tool for long-term use in diverse settings.

### Radiomics and Deep Features

Deep learning-based features can be extracted from MRIs using deep neural networks trained in supervised settings such as tumor segmentation, self-supervised learning methods (Chen et al., 2020; Zbontar et al., 2021), and unsupervised architectures such as autoencoders (Biggs et al., 2023). These features are not readily interpretable and cannot be easily validated yet can capture information that may be useful in training prognostic and diagnostic models that are missed by interpretable features. We believe radiomics can complement deep features and provide additional value as they are easier to validate and are interpretable, providing rich and useful descriptions of disease processes that may be inherently useful clinically and building trust in ML models’ decisions.

## Information Sharing Statement

The source code for ALFE is available in the following public repository: https://github.com/reghbali/pyalfe. The models used by ALFE are available in the following Hugging Face public repository: https://huggingface.co/reghbali/pyalfe-models. Additionally, the Python package for ALFE can be found onPyPI: https://pypi.org/project/pyalfe/.

## Supplementary Information

Below is the link to the electronic supplementary material.

Supplementary file 1 (pdf 109 KB)

## Data Availability

Data is provided within the manuscript or supplementary information files. Models and images are accessible through open-access repositories on github.com and huggingface.co.

## Footnotes

1 Note that a small fraction of the lesions (less than 10%) overlap CSF which is due to tissue segmentation error.
