# Supplementary material for: Automated Lesion and Feature Extraction Pipeline for Brain MRIs with Interpretability
Source: Neuroinformatics. 2025 Jan 9;23(1):2. doi: 10.1007/s12021-024-09708-z (PMC11717894; doi:10.1007/s12021-024-09708-z)
Supplement: Supplementary file 1 — (pdf 109 KB) [file 12021_2024_9708_MOESM1_ESM.pdf]

Automated Lesion and Feature Extraction Pipeline for Brain MRIs with Interpretability

Reza Eghbali[1,3*], Pierre Nedelec[1], David Weiss[5], Radhika Bhalerao[1], Long Xie[6], Jeffrey D. Rudie[4], Chunlei Liu[2], Leo P. Sugrue[1], Andreas M. Rauschecker[1]

[1]Department of Radiology and Biomedical Imaging, University of California, San Francisco.
[2]Department of Electrical Engineering and Computer Sciences, University of California, Berkeley.
[3]Berekely Institute for Data Science, University of California, Berkeley.
[4]Department of Radiology, University of California, San Diego.
[5]Department of Biomedical Engineering, Georgia Institute of Technology.
[6]Siemens Healthineers.

*Corresponding author(s). E-mail(s): eghbali@berkeley.edu;
Contributing authors: pierre.nedelec@ucsf.edu; dweiss38@gatech.edu; radhika.bhalerao@ucsf.edu; long.xie@siemens-healthineers.com; jeff.rudie@gmail.com; chunlei.liu@berkeley.edu; leo.sugrue@ucsf.edu; andreas.rauschecker@ucsf.edu;

Supplementary Material

Feature	value
total brain volume (mm^3)	1248108
total ventricles volume (mm^3)	35796
volume of background (mm^3)	3319025
volume of csf (mm^3)	325795
volume of cortical gray matter (mm^3)	433235
volume of white matter (mm^3)	415505
volume of deep gray matter (mm^3)	38540
volume of brain stem (mm^3)	21968
volume of cerebellum (mm^3)	151962
volume of Frontal (mm^3)	504398
volume of Parietal (mm^3)	245407
volume of Occipital (mm^3)	151885
volume of Temporal (mm^3)	229425
volume of AnteriorTemporal (mm^3)	70165
volume of MiddleTemporal (mm^3)	118049
volume of PosteriorTemporal (mm^3)	41211
volume of Parietal Occipital (mm^3)	397292
volume of CorpusCallosum (mm^3)	30245
volume of CorpusCallosum Rostrum (mm^3)	5134
volume of CorpusCallosum Genu (mm^3)	5803
volume of CorpusCallosum Body (mm^3)	10986
volume of CorpusCallosum Isthmus (mm^3)	2732
volume of CorpusCallosum Splenium (mm^3)	5590
volume of CSF (mm^3)	301750
volume of Cortical Gray Matter (mm^3)	434435
volume of White Matter (mm^3)	413317
volume of Deep Gray Matter (mm^3)	47373
volume of Brain Stem (mm^3)	23549
volume of Cerebellum (mm^3)	134312

Table 1 Brain volumetric features for the PCNSL case generated by the pipeline run with the image processing implementation based on C3D and the image registration implementation based on ANTsPy.

Feature	T1Post Lesion	FLAIR Lesion
total lesion volume (mm^3)	1614	16857.2
lesion volume in csf (mm^3)	0	101
lesion volume in cortical gray matter (mm^3)	0	91
lesion volume in white matter (mm^3)	1603	15211.2
lesion volume in deep gray matter (mm^3)	0	27
lesion volume in brain stem (mm^3)	0	0
lesion volume in cerebellum (mm^3)	0	0
relative T1 signal	0.9	0.9
relative T1Post signal	1.5	1.0
relative FLAIR signal	1.3	1.5
relative T2 signal	1.3	1.4
relative ADC signal	0.9	1.3
mean ADC signal ($10^{-6}mm^2/s$)	766.5	1022.7
median ADC signal ($10^{-6}mm^2/s$)	728.5	988.3
five percentile ADC signal ($10^{-6}mm^2/s$)	626.1	708.3
ninety five percentile ADC signal ($10^{-6}mm^2/s$)	1032.7	1453.4
relative SWI signal	1.0	1.1
relative CBF signal	1.1	0.8
mean CBF signal (mL/100g/min)	65.4	48.2
median CBF signal (mL/100g/min)	67.6	46.9
five percentile CBF signal (mL/100g/min)	44.5	29.3
ninety five percentile CBF signal (mL/100g/min)	80.2	72.0
enhancement	1.9	1.3
average dist to ventricles (voxels)	9.2	9.3
minimum dist to Ventricles (voxels)	0.9	0
lesion volume in Frontal (mm^3)	1614	14644
percentage volume in Frontal	100	86.9
lesion volume in Parietal (mm^3)	0	1560
percentage volume in Parietal	0	9.3
lesion volume in Occipital (mm^3)	0	448
percentage volume in Occipital	0	2.7
lesion volume in Temporal (mm^3)	0	205.2
percentage volume in Temporal	0	1.2
lesion volume in CorpusCallosum (mm^3)	210	1655.8
percentage volume in CorpusCallosum	13.0	9.8
number of lesions	1	30
largest lesion volume (mm^3)	1614	10419

Table 2 Summary lesion features for the PCNSL case generated by the pipeline run with the image processing implementation based on C3D and the image registration implementation based on ANTsPy.

Feature	Lesion 0	Lesion 1	Lesion 2	Lesion 3
total lesion volume (mm^3)	10419	3279.8	955.2	493.5
lesion volume in csf (mm^3)	47.5	10.2	23	6
lesion volume in cortical gray matter (mm^3)	5.8	85.2	0	0
lesion volume in white matter (mm^3)	9818.8	2688.2	773.5	391.8
lesion volume in deep gray matter (mm^3)	11.5	3.2	0	0
lesion volume in brain stem (mm^3)	0	0	0	0
lesion volume in cerebellum (mm^3)	0	0	0	0
relative T1 signal	0.9	0.8	0.9	0.8
relative T1Post signal	1.0	0.9	0.9	0.8
relative FLAIR signal	1.4	1.5	1.7	1.8
relative T2 signal	1.4	1.4	1.5	1.5
relative ADC signal	1.2	1.4	1.5	1.5
mean ADC signal ($10^{-6}mm^2/s$)	959.3	1136.9	1180.0	1199.4
median ADC signal ($10^{-6}mm^2/s$)	934.0	1096.8	1118.9	1192.4
five percentile ADC signal ($10^{-6}mm^2/s$)	685.0	857.7	913.0	953.7
ninety five percentile ADC signal ($10^{-6}mm^2/s$)	1361.4	1577.8	1686.2	1466.0
relative SWI signal	1.0	1.0	1.1	1.1
relative CBF signal	0.8	0.7	0.5	0.6
mean CBF signal (mL/100g/min)	51.4	41.5	31.9	38.0
median CBF signal (mL/100g/min)	49.6	39.1	31.0	37.3
five percentile CBF signal (mL/100g/min)	34.6	26.8	26.2	31.9
ninety five percentile CBF signal (mL/100g/min)	73.9	64.3	41.4	47.4
enhancement	1.3	1.2	1.2	1.2
average dist to ventricles (voxels)	9.8	8.0	3.7	2.5
minimum dist to Ventricles (voxels)	0	0	0	0
lesion volume in Frontal (mm^3)	10326.2	3279.2	0	0
percentage volume in Frontal	99.1	100.0	0	0
lesion volume in Parietal (mm^3)	92.8	0.5	689.8	209
percentage volume in Parietal	0.9	0.0	72.2	42.4
lesion volume in Occipital (mm^3)	0	0	185.5	187
percentage volume in Occipital	0	0	19.4	37.9
lesion volume in Temporal (mm^3)	0	0	80	97.5
percentage volume in Temporal	0	0	8.4	19.8
lesion volume in CorpusCallosum (mm^3)	1197.5	279.5	85.8	43.5
percentage volume in CorpusCallosum	11.5	8.5	9.0	8.8

Table 3 Individual lesion features for the four largest FLAIR lesions in the PCNSL case generated by the pipeline run with the image processing implementation based on C3D and the image registration implementation based on ANTsPy.
